# Synthesis and Investigation of a Radioiodinated F3 Peptide Analog as a SPECT Tumor Imaging Radioligand

**DOI:** 10.1371/journal.pone.0022418

**Published:** 2011-07-19

**Authors:** Mahaveer S. Bhojani, Rajesh Ranga, Gary D. Luker, Alnawaz Rehemtulla, Brian D. Ross, Marcian E. Van Dort

**Affiliations:** 1 Department of Radiation Oncology, University of Michigan, Ann Arbor, Michigan, United States of America; 2 Department of Radiology, University of Michigan, Ann Arbor, Michigan, United States of America; 3 Department of Microbiology and Immunology, University of Michigan, Ann Arbor, Michigan, United States of America; 4 Center for Molecular Imaging, University of Michigan, Ann Arbor, Michigan, United States of America; National Institute of Health, United States of America

## Abstract

A radioiodinated derivative of the tumor-homing F3 peptide, (*N*-(2-{3-[^125^I]Iodobenzoyl}aminoethyl)maleimide-F3Cys peptide, [^125^I]IBMF3 was developed for investigation as a SPECT tumor imaging radioligand. For this purpose, we custom synthesized a modified F3 peptide analog (F3Cys) incorporating a C-terminal cysteine residue for site-specific attachment of a radioiodinated maleimide conjugating group. Initial proof-of-concept Fluorescence studies conducted with AlexaFluor 532 C_5_ maleimide-labeled F3Cys showed distinct membrane and nuclear localization of F3Cys in MDA-MB-435 cells. Additionally, F3Cys conjugated with NIR fluorochrome AlexaFluor 647 C_2_ maleimide demonstrated high tumor specific uptake in melanoma cancer MDA-MB-435 and lung cancer A549 xenografts in nude mice whereas a similarly labeled control peptide did not show any tumor uptake. These results were also confirmed by *ex vivo* tissue analysis. No-carrier-added [^125^I]IBMF3 was synthesized by a radioiododestannylation approach in 73% overall radiochemical yield. *In vitro* cell uptake studies conducted with [^125^I]IBMF3 displayed a 5-fold increase in its cell uptake at 4 h when compared to controls. SPECT imaging studies with [^125^I]IBMF3 in tumor bearing nude mice showed clear visualization of MDA-MB-435 xenografts on systemic administration. These studies demonstrate a potential utility of F3 peptide-based radioligands for tumor imaging with PET or SPECT techniques.

## Introduction

Recent advances in cellular and molecular biology have made much progress in unraveling the molecular footprints of tumorigenesis by identifying abnormalities in signal transduction pathways and gene alterations associated with cancer initiation and progression. Although a number of tumor biomarkers have been identified and their roles construed, the true impact of these advances is still not apparent in the clinic. This is partly due to the fact that clinical diagnosis and outcome of therapy is still determined by assessment of gross structural measurements obtained several months after therapy administration. Inability to dynamically monitor biomarker activity within the tumor and its microenvironment is a major impediment to the improvement of overall patient survival rates. PET and SPECT imaging modalities have been proven to be clinically relevant in the diagnosis and monitoring of therapeutic response of many cancers [1], [2]. Accordingly, there is a an urgent need for improved tumor-specific PET and SPECT radioligands that home to cancerous tissue and allow detection and diagnosis of cancer.

F3 peptide (KDEPQRRSARLSAKPAPPKPEPKPKKAPAKK), a 31 amino acid fragment of HMGN2 protein identified by Ruoslahti's group using *in vivo* phage display techniques, has been shown to bind to tumor and endothelial cells in tumor blood vessels [3]. Subsequent studies showed that F3 peptide binds to nucleolin at cell surface and is transported to nucleus in a nucleolin-dependent manner [4]. Nucleolin is a highly conserved nuclear protein that is involved in ribosome maturation [5], [6]. It also acts as a shuttle protein that transports cargo between cytoplasm and nucleus [7], [8]. In normal cells, nucleolin resides in the nucleus, however in tumor cells it is reported to shuttle between the cell surface and nucleus [9], [10]. Because of its distinct localization on tumor cell membrane, specific antagonists of cell surface nucleolin are being actively investigated for tumor targeting [4], [11].

We have previously shown that F3 peptide aids in targeting and retention of multifunctional nanoparticles to tumor and its milieu [12], [13].The tumor-homing and internalizing properties of F3 peptide make it an attractive candidate as a scaffold for development of novel radioligands for PET or SPECT tumor imaging. Herein, we report the development of an [^125^I]-labeled F3 peptide conjugate as a SPECT tumor imaging radioligand and its initial proof-of concept preclinical mouse imaging studies.

## Results and Discussion

### Synthesis of Fluorescent-tagged F3Cys peptide

**Figure 1:**
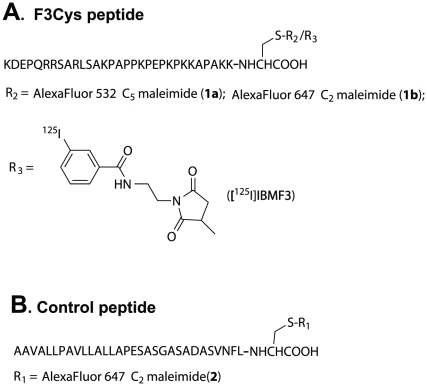
Fluorescent- and [^125^I]-labeled F3Cys Analogs. Structures of fluorescent and radioiodinated F3Cys and control peptide analogs investigated in this study (for details see text).

Common methods for peptide labeling utilize the conjugation of prosthetic groups such as succinimidyl esters to the ε-amino group of lysine residues [14]. However, site-specific labeling of F3 peptide by this approach is problematic since it contains a total of 9 lysine residues. Consequently, our strategy for site-specific functionalization focused on labeling at the free thiol of a suitable cysteine-modified F3 peptide analog. Thiol-specific reagents such as maleimides have been shown to display high chemoselectivity as compared to amino or carboxylate-reactive reagents [15], [16]. Accordingly, we custom synthesized a modified F3 peptide analog incorporating a cysteine residue at the C-terminus (F3Cys) for site specific coupling to a radioiodinated maleimide conjugating group. We chose a *meta*-[^125^I]iodobenzamide group tethered to a maleimide as our labeled conjugating moiety since meta-substituted aromatic radioiodine labels are known to demonstrate high stability towards *in vivo* metabolic deiodination [17], [18]. AlexaFluor 532 C_5_ maleimide-labeled F3Cys (AF532-F3Cys (**1a**), Figure 1A) was synthesized to investigate the sub-cellular distribution of F3Cys. We also labeled F3Cys with the Near Infrared (NIR) fluorochrome AlexaFluor 647 C_2_ maleimide (AF647-F3Cys (**1b**); Figure 1A) for *in vivo* optical imaging studies. To demonstrate that the tumor localization of F3Cys conjugate was specific to the F3 peptide sequence, the corresponding AlexaFluor 647 C_2_ maleimide conjugate of a control peptide (AF647-Control (**2**), Figure 1B) incorporating cysteine at the C-terminus was also synthesized.

### *In vitro* Cell Studies with AlexaFluor 532 C_5_ maleimide-labeled F3Cys (AF532-F3Cys)

**Figure 2:**
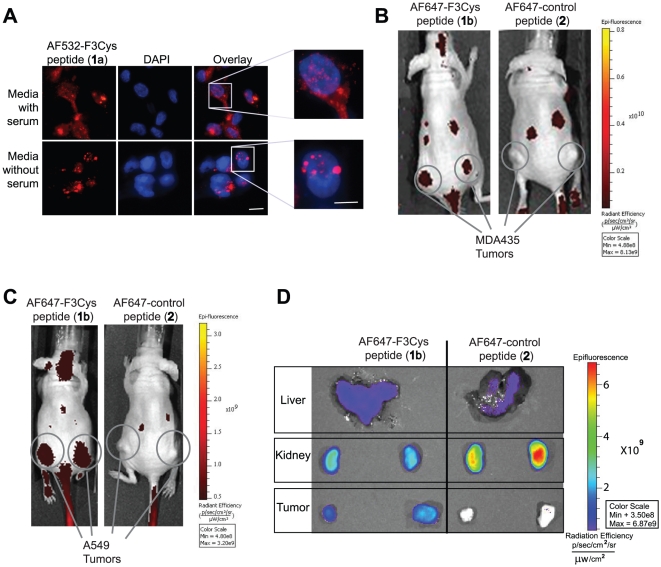
*In vitro* and *in vivo* optical imaging using Fluorescent-labeled F3Cys peptides. MDA-MB-435 cells, in optically clear bottom dishes, cultured in either serum free or serum containing media were stained with AF532-F3Cys, counterstained with DAPI and monitored under a fluorescent microscope (**A**). Mice bearing MDA-MB-435 (**B**) or A549 (**C**) xenografts were injected i.v. via the tail vein with either AF647-F3Cys (**1b**) or AF647-Control peptide (**2**). Fluorescence images were acquired over time and a representative image obtained at 2 h is shown (**B** and **C**). *Ex vivo* fluorescence imaging of tumor, kidney and liver harvested 2 h after AF647-F3Cys peptide injection in animals bearing A549 tumor xenograft (**D**).

When MDA-MB-435 cells were grown in media containing 10% serum and stained with AF532-F3Cys, cell surface and nuclear staining of F3Cys was observed. On the other hand, serum starved cells showed predominantly nuclear staining without significant membrane staining suggesting that F3Cys localizes to cell surface in actively growing cells (Figure 2A). This pattern of staining has been previously demonstrated for F3 peptide and corresponds to the subcellular distribution of nucleolin in serum-starved as well as proliferating cells [3].

### *In Vivo* Studies with Fluorescent-labeled F3Cys and Control peptides

Nude mice with MDA-MB-435 xenografts were injected i.v. with AF647-F3Cys or AF647-Control peptide and imaged using an *in vivo* fluorescence imaging system. Tumor specific uptake at 2 h was observed only with AF647-F3Cys but not with AF647-Control peptide (Figure 2B). Tumor retention of AF647-F3Cys was also observed at 24 h post injection *albeit* at lower levels (data not shown). In addition to tumor, there are other sites which show fluorescence signal in the images. We believe that this likely represent kidney uptake (see below). Further, to validate that F3 imaging of tumor is also applicable to other tumor models, we imaged A549 lung tumor xenografts in nude mice. Similar to the MDA-MB-435 *in vivo* imaging studies, the A549 tumor also selectively retained AF647-F3Cys, while the AF647-Control peptide did not display any tumor localization (Figure 2C). These studies confirm that F3Cys retains the tumor-localizing properties of F3 peptide. Additionally, parallel imaging studies with the labeled control peptide confirm that the tumor localization of the AF647-F3Cys was specific to the F3 peptide sequence.

To validate the fluorescence imaging data, animals were sacrificed and tumors and excretory organs were harvested for *ex vivo* fluorescence analysis. In addition to tumors, a high fluorescence signal was observed in kidney and liver (Figure 2D). Kidney and liver uptake was also seen with animals injected with AF647-Control peptide but no tumor specific fluorescence was observed. This suggests that non-tumor signal observed in whole body imaging likely represents kidney and liver uptake.

### Synthesis of radioiodinated F3Cys peptide ([^125^I]IBMF3)

**Figure 3:**
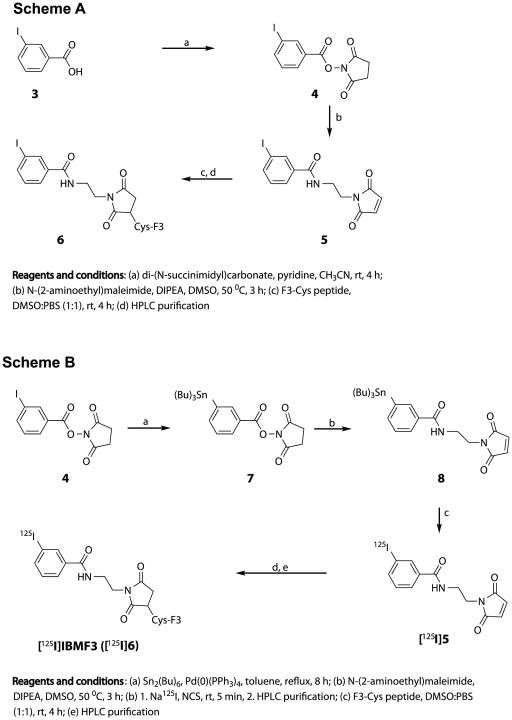
Synthesis of [^125^I]IBMF3. Chemical synthetic approach for preparation of non-radioactive IBMF3 (**6**) (**Scheme A**) and [^125^I]IBMF3 (**Scheme B**) (See text for details).

The structure of the target radioligand ([^125^I]IBMF3) chosen for synthesis and investigation is shown in Scheme B (Figure 3). Prior to radioligand synthesis, the non-radioactive analog (IBMF3, **6**) was synthesized for method optimization and structure characterization by the approach shown in Scheme A (Figure 3). Initially, conversion of 3-iodobenzoic acid (**3**) to the corresponding succinimidyl ester **4** was achieved in 97% yield using di-(*N*-succinimidyl)carbonate in pyridine/CH_3_CN. Treatment of **4** with *N*-(2-aminoethyl)maleimide and DIPEA at moderately elevated temperatures provided the non-radioactive iodinated maleimide conjugate reagent **5** in 58% yield. Conjugation of **5** to F3Cys peptide and subsequent HPLC purification of the non-radioactive iodinated peptide conjugate **6** was conducted as described for the synthesis of the fluorescence-tagged ligands.

Synthesis of radioiodinated F3Cys peptide ([^125I^]IBMF3) was conducted using a radioiododestannylation approach (Scheme B). The 3-(*tri*-n-butylstannyl)maleimide analog **8** was obtained from the *N*-succinimidyl-3-(*tri*-n-butylstannyl)benzoate analog **7** using the approach described for the synthesis of **5** (Scheme A). Radioiododestannylation of intermediate **8** with Na^125^I using NCS as oxidizing agent provided **[^125^I]5** in 89% radiochemical yield and >99% radiochemical and chemical purity after HPLC purification. The radioiodinated product was well resolved from the *tri*-n-butylstannyl starting material (Rt>25 min) under the HPLC conditions. Conjugation of **[^125^I]5** to F3Cys peptide was carried out in a mixture of DMSO and PBS buffer at 4°C. Radioiodinated F3Cys peptide conjugate [^125^I]IBMF3 was obtained in 73% overall radiochemical yield and >99% radiochemical and chemical purity after HPLC purification (Method II). Under these HPLC conditions, [^125^I]IBMF3 was well separated from F3Cys peptide (Rt = 16.1 min). The average specific activity of [^125^I]IBMF3 was determined to be 1793±164 Ci/mmol (N = 3). The radioligand which was formulated in PBS∶EtOH [95∶5] was stable for at least 1 week when stored at 5°C (<2% radiolytic decomposition by radio-HPLC analysis).

### *In Vitro* Cell Uptake Studies with [^125^I]IBMF3

**Figure 4:**
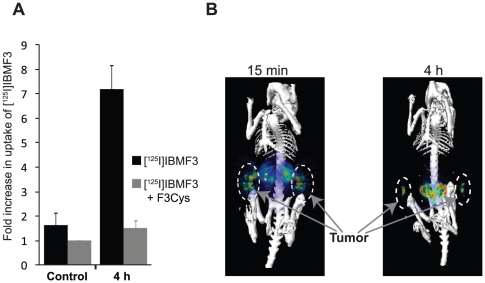
*In vitro* cell uptake and *in vivo* SPECT imaging with [^125^I]IBMF3. (**A**) MDA-MB-435 cells were incubated with [^125^I]IBMF3 either alone or in the presence of F3Cys for 0 h or 4 h and uptake of [^125^I]IBMF3 was measured. To determine non-specific binding cells in control group were treated with [^125^I]IBMF3, the media removed immediately after addition of the radioligand and processed as for the experimental group. All experiments were performed in triplicate and repeated at least thrice. The data presented here is an average of radioactivity counts per well +/− SD. (**B**) Mice bearing MDA-MB-435 xenografts were injected i.v. via the tail vein with [^125^I]IBMF3 and animals were serially imaged post injection. Representative images obtained at 15 min and 4 h post-injection are shown.

The cellular uptake of [^125^I]IBMF3 was determined by incubating MDA-MB-435 cells with [^125^I]IBMF3 for 4 h in presence or absence of F3Cys. An almost 5-fold increase in [^125^I]IBMF3 uptake was observed at 4 h when compared to controls (Figure 4A). Further, competition with unlabeled F3Cys peptide reduced the uptake of [^125^I]IBMF3 to levels seen in controls, confirming the specificity of its cell uptake.

### *In Vivo* SPECT Imaging Studies

SPECT imaging studies conducted following i.v. administration of [^125^I]IBMF3 to nude mice bearing MDA-MB-435 tumor xenografts showed distinct uptake of radioactivity in tumor as early as 15 min post-injection (Figure 4B). Non-target accumulation of radioactivity was mainly in kidney and bladder between 15 min (Figure 4B) and 2 h (data not shown) suggesting that renal elimination was the major excretory pathway. At 4 h, kidney and bladder were not visualized likely due to voiding of urine. It is possible that the presence of urine radioactivity in kidney and bladder, at the early time intervals, could have contributed to an overestimation of tissue radioactivity uptake in these organs in the images. Tumors were also visualized in the SPECT images at 4 h although at lower levels compared to the 15 min and 2 h images. Tumor uptake of radioactivity displayed substantial clearance at later time intervals (>4 h) resulting in poor image visualization of tumor. Intestinal radioactivity was clearly apparent in the 4 h images, which is most likely due to a shift in radioactivity clearance from the renal to hepatobiliary excretion pathway at later time intervals. Moderate radioactivity uptake was seen in thyroid indicating that [^125^I]IBMF3 is relatively stable towards *in vivo* deiodination.

### Biodistribution Studies

**Figure 5:**
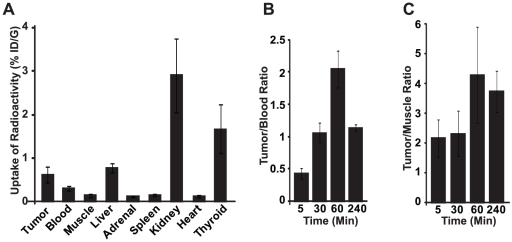
Tissue biodistribution of [^125^I]IBMF3 in MDA MB-435 tumor bearing mice. Mice (n = 4 per time interval) were injected via tail vein with [^125^I]IBMF3 and tissues were harvested for radioactivity uptake. (**A**) Tissue distribution at 60 min post injection and reported as percent injected dose per gram of tissue (%ID/g). (**B**) and (**C**) represent the ratio of radioactivity in tumor to blood and tumor to muscle respectively at various time intervals. All the data are computed as mean +/− SEM.

The uptake of radioactivity in selected tissues was determined following intravenous administration of [^125^I]IBMF3 to MDA-MB-435 tumor-bearing athymic nude mice. Mice were injected intravenously with 7.8–10 µCi of [^125^I]IBMF3 and sacrificed at designated time intervals (5 min, 30 min, 1 h, and 4 h post-injection). These results are summarized in Figure 5 and complete biodistribution results are presented in Supplementary data (Table S1). [^125^I]IBMF3 displayed high radioactivity accumulation in kidney and to a lesser extent in liver at the early time intervals. Kidney uptake of radioactivity (5.68%ID/g and 10.8% ID/g at 5 and 30 min respectively post-injection) was the highest among all sampled tissues confirming that renal elimination is the major excretory pathway for the radioligand (Table S1). In tumors, radioactivity levels peaked at 30 min post-injection (1.05% ID/g). Although initial blood levels of radioactivity were moderately high (0.84% ID/g at 30 min) this tissue displayed a >60% radioactivity clearance between 30 and 60 min resulting in tumor-to-muscle and tumor-to-blood ratios of 4.3 and 2.1, respectively, at 60 min post-injection (Figure 5B and 5C). Moderate radioactivity uptake was seen in thyroid (maximal uptake of 4% ID/g at 4 h) indicating that the radioligand is relatively stable towards *in vivo* deiodination (Supplementary data). As expected due to its highly hydrophilic nature, [^125^I]IBMF3 displayed very low extraction into brain (0.03%ID/g at 5 min post-injection; data not shown). No appreciable radioactivity uptake was seen in other sampled tissues such as spleen, adrenal and heart.

### Radioligand Metabolism

The metabolic stability of [^125^I]IBMF3 in blood and urine was investigated at 5 min post-injection in tumor-bearing athymic nude mice by HPLC analysis of the relative amounts of parent radioligand and radiometabolites. Analysis of plasma samples showed that 24.6% of the total radioactivity at 5 min post-injection was associated with intact [^125^I]IBMF3. The remaining radioactivity consisted primarily of two hydrophobic radiometabolites representing 47% and 18% of the total plasma radioactivity. Free radioiodide (^125^I) accounted for 6.4% of the plasma radioactivity. In contrast, the parent radioligand accounted for 42% of the total radioactivity present in mouse urine samples at this time interval. The remaining radioactivity consisted of three major hydrophobic radiometabolites representing 28%, 6.8% and 10% of the total. Thus, despite its limited availability in blood as the intact radioligand at early time points, [^125^I]IBMF3 still provided clear visualization of tumors in the SPECT images up to 4 h post-injection. This is possibly due to its high in vivo tumor affinity which results in its rapid sequestration and retention within the tumor cells in a nucleolin dependent manner.

### Conclusion

Our studies show a potential utility of the tumor-homing F3 peptide as a radiodiagnostic for PET or SPECT tumor imaging. Tumor homing peptides have several advantages over antibodies or large proteins due to minimal non-specific uptake by the reticuloendothelial system and the low likelihood of an immune response because of their smaller size. Additionally, PET and SPECT imaging approaches offer unique advantages including, high sensitivity, as well as, tomographic, deep tissue and repetitive imaging capabilities. Further, these imaging techniques are directly translatable to clinical application. In previously reported preclinical studies, F3 peptide has been utilized to target a payload to tumor sites. These include theranostic nanoparticles (reported by our laboratory [12], [13]), magnetic nanoworms [19], quantum dots harboring siRNA [20], hydrogel-based nanoparticles harboring therapeutic agents [21]–[23], fluorescent probes [3], [12] and transduction baculovirus to tumors [24]. Additionally, Drecoll et al recently demonstrated the use of F3 peptide conjugated with ^213^Bi or ^68^Ga for application for tumor radiotherapy and PET imaging, respectively, in a mouse model of peritoneal carcinomatosis [25]. In their reported tissue biodistribution studies, i.p.-administered [^213^Bi]-labeled F3 peptide displayed superior tumor uptake and tumor-to-blood and tumor-to-muscle ratios in their tumor model in comparison to our results with i.v. administered [^125^I]IBMF3 in the MDA-MB-435 tumor xenograft mouse model. The enhanced tumor uptake of radioactivity seen in these studies could be due to high local radiotracer availability in the tumor vicinity as a consequence of intraperitoneal administration.

The high renal clearance of [^125^I]IBMF3 and its subsequent moderate levels of tumor uptake following systemic administration, could pose a challenge to SPECT imaging of deep tissues in close proximity to these organs. High renal excretion of radioactivity was also seen by Drecoll *et al* [25] in their studies with [^213^Bi]-conjugated F3 peptide. It is possible that a structurally modified version of this radioligand may offer more suitable *in vivo* pharmacokinetics including reduced renal clearance and consequently higher tumor uptake for SPECT imaging. One potential approach to retard renal clearance would be to incorporate a more hydrophobic linker for radiolabel attachment to F3Cys peptide. Alternatively, modifications such as the use of D amino acids, glycosylation or the masking of C or N termini could also be utilized for improvement of its pharmacokinetic properties as previously reported for RGD or other peptides [26]–[28]. Studies in this regard are currently underway in our laboratory.

## Materials and Methods

### Chemicals and Radiochemicals

Cysteine-modified F3 peptide (KDEPQRRSARLSAKPAPPKPEPKPKKAPAKKC; F3Cys) was custom synthesized by PolyPeptide Laboratories, Torrance, CA using standard solid-phase fluorenylmethoxycarbonyl (Fmoc) chemistry techniques. Control peptide (AAVALLPAVLLALLAPESASGASADASVNFLC) was synthesized by Peptide 2.0 Corp., Chantilly, VA, and Alexa Fluor 647 C_2_ maleimide was obtained from Invitrogen Corp., Carlsbad, CA. All other chemical reagents were obtained from Aldrich Chemical Co., Milwaukee, WI. Sodium [^125^I]iodide was obtained from MDS Nordion Inc. (Ottawa, Canada) as a no-carrier-added solution in aqueous 0.01 N NaOH (pH 10–12).

### Instrumentation and Analyses

Melting points were determined with a Thomas-Hoover melting point apparatus and are uncorrected. Thin-layer chromatography (TLC) was performed using Analtech silica gel GF Uniplates (250 micron). TLC plates were visualized after development with either ultraviolet (UV) light or by spraying with phosphomolybdic acid reagent with subsequent heating. ^1^H NMR spectra were recorded on a Varian INOVA instrument operating at 400 MHz using CDCl_3_, or DMSO-*d*_6_ as solvent and tetramethylsilane (TMS) as internal standard. Chemical shifts (δ) and coupling constants (*J*) are reported in parts per million (ppm) and Hertz (Hz), respectively. Compound elemental analysis data were obtained at the Department of Chemistry, University of Michigan. High resolution mass spectral analyses were performed at the Department of Chemistry, University of Michigan, using either a VG-70-250-S mass spectrometer for electron impact (EI) and chemical ionization (DCI) modes, a Waters Autospec Ultima instrument with an electrospray interface for electrospray ionization (ESI) mode or a Waters Tofspec-2E run in reflectron mode. Elemental analyses were conducted by Atlantic Microlab Inc., Norcross, GA. HPLC was performed using a Waters Breeze HPLC System (Waters Corporation, Milford, MA) equipped with a Waters 2487 Dual Wavelength Absorbance Detector. Radioactivity was monitored with a Bioscan Flow Count FC-3300 NaI/PMT Radiodetector (Bioscan, Inc., Washington, DC) equipped with a 1.5″×1.5″ NaI(Tl) crystal. HPLC analysis and purification were conducted at ambient temperature on a Waters Sunfire C-18 column (4.6×250 mm), 5 µ particle, with 0.1% TFA in H_2_O (A) and 0.1% TFA in CH_3_CN (B) solvent mixtures with UV absorbance (215 and 254 nm) and radioactivity detection. HPLC runs were conducted using either of the following methods: Method I [Initial gradient elution from 90% A to 5% A over 25 min; flow rate of 1 mL/min]; Method II [Initial gradient elution from 92% A to 84% A over 32 min followed by 84% A to 68% A over 8 min; flow rate of 1.5 mL/min]. Radioactivity measurements were obtained with a Capintec CRC-15W Radioisotope Dose Calibrator (Ramsey, NJ) and specific activity estimates were determined from a standard curve relating mass to UV absorbance peak area.

### General Method for Synthesis of Alexa Fluor Maleimide-labeled Peptide Conjugates (1a, 1b and 2)

The method of preparation of compound **1b** is described as an example. A solution of Alexa Fluor 647 C_2_ maleimide reagent (1 mg, 0.77 µmol) in DMSO (150 µl) was added to a stirred solution of F3Cys peptide (0.77 µmol) in 150 µL of DMSO. The mixture was stirred in the dark under a nitrogen atmosphere at ambient temperature for 18 h, at which point, HPLC analysis (Method II) showed completion of reaction. HPLC purification afforded the fluorescence-tagged peptides in 69–83% yields. Rt (**1a**) = 34.3 min; Rt (**1b**) = 35.5 min; Rt (**2**) = 26.1 min.

### *N*-Succinimidyl-3-iodobenzoate (4)

The title compound was synthesized in 97% yield from 3-iodobenzoic acid (**3**) using a general method described by Koziorowski et al [29]; mp 147–148°C. ^1^H NMR (DMSO-d_6_): δ 8.36 (t, 1H, *J* = 1.6 Hz), 8.23 – 8.21 (m, 1H), 8.13 – 8.10 (m, 1H), 7.47 (t, 1H, *J* = 7.9 Hz), 2.91 (s, 4H). HRMS [EI, 70 eV]: Calcd for C_11_H_8_NO_4_I: 344.9498 [M^+^]. Found: 344.9492. Anal. (C_11_H_8_NO_4_I): C, H, N.

### *N*-(2-{3-Iodobenzoyl}aminoethyl)maleimide (5)

A solution of **4** (51 mg, 0.1 mmol) in anhydrous DMSO (0.6 mL) was treated sequentially with *N*-(2-aminoethyl)maleimide trifluoroacetate salt (26 mg, 0.1 mmol) and (i-Pr)_2_NEt (0.02 mL, 0.11 mmol) and stirred at 50–55°C for 3 h under an argon atmosphere. Brine was added to the reaction, the mixture was extracted with EtOAc and the combined organic layers were washed with brine, H_2_O, and dried (Na_2_SO_4_). The crude product was purified by flash chromatography on silica gel (20–40% EtOAc in hexanes) to give the title compound in 58% yield as a white crystalline solid: mp 171–172°C; ^1^H NMR (DMSO-d_6_): δ 8.65 (t, 1H, *J* = 5.9 Hz), 8.07 (t, 1H, *J* = 1.7 Hz), 7.89 – 7.86 (m, 1H), 7.75 – 7.72 (m, 1H), 7.26 (t, 1H, *J* = 7.8 Hz), 7.02 (s, 2H), 3.57 (t, 2H, *J* = 5.6 Hz), 3.41 – 3.37 (m, 2H). HPLC (Method I; R_t_ = 15.7 min) HRMS [ESI with Na^+^ added]: Calcd for C_13_H_11_N_2_O_3_INa: 392.9712 [M+Na^+^]. Found: 392.9707. Anal. (C_13_H_11_N_2_O_3_I): C, H, N.

### Synthesis of Iodinated F3-Cys Peptide Conjugate (6, IBMF3)

The title compound was prepared as described above by addition of a solution of **5** (0.9 mg, 2.2 µmol) in DMSO (50 µl) to a stirred solution of F3-Cys peptide (7 mg, 1.98 µmol) in PBS buffer (100 µL, pH 7.0). The product was purified by HPLC (Method II; Rt = 35.4 min) to afford **6** in 76% yield. HRMS {MALDI TOF}: Calcd for C_168_H_279_N_52_O_45_IS: 3906. Found: 3907 [MH+].

### *N*-Succinimidyl-3-(*tri*-n-butylstannyl)benzoate (7)

The title compound was obtained in 63% yield as a colorless oil from compound **4** using the general approach of Dekker and coworkers [30]. ^1^H NMR (CDCl_3_): δ 8.19 (s, 1H), 8.07 – 8.04 (m, 1H), 7.77 – 7.75 (m, 1H), 7.44 (t, 1H, *J* = 7.5 Hz), 2.91 (s, 4H), 1.57 – 1.50 (m, 6H), 1.37 – 1.28 (m, 6H), 1.12 – 1.08 (m, 6H), 0.93 – 0.87 (m, 9H). HRMS [ESI with Na^+^ added]: Calcd for C_23_H_35_NO_4_Sn: 532.1486 [M+Na^+^]. Found: 532.1509.

### *N*-(2-{3-(*tri*-n-Butylstannyl)benzoyl}aminoethyl)maleimide (8)

A solution of **7** (51 mg, 0.1 mmol) in anhydrous DMSO (0.6 mL) was treated sequentially with *N*-(2-aminoethyl)maleimide trifluoroacetate salt (26 mg, 0.1 mmol) and (i-Pr)_2_NEt (0.02 mL, 0.11 mmol) and stirred at 50–55°C for 3 h under argon. Brine was added to the reaction, the mixture was extracted with EtOAc and the combined organic layers were washed with brine, H_2_O, and dried (Na_2_SO_4_). The crude product was purified by flash chromatography on silica gel (20–40% EtOAc in hexanes) to give the title compound in 53% yield as an off-white amorphous solid. ^1^H NMR (CDCl_3_): δ 7.86 - 7.85 (m, 1H), 7.72 – 7.63 (m, 1H), 7.59 – 7.52 (m, 1H), 7.39 – 7.35 (m, 1H), 6.73 (s, 2H), 6.66 (br t, 1H), 3.86 - 3.83 (m, 2H), 3.69 – 3.65 (m, 2H), 1.57 – 1.50 (m, 6H), 1.37 – 1.28 (m, 6H), 1.11 – 1.02 (m, 6H), 0.94 – 0.85 (m, 9H). (R_t_ = 34.12 min; 90∶10 to 5∶95) HRMS [ESI with Na^+^ added]: Calcd for C_25_H_38_N_2_O_3_SnNa: 557.1802 [M+Na^+^]. Found: 557.1796. Anal. (C_25_H_38_N_2_O_3_Sn): C, H, N.

### Synthesis of [^125^I]-labeled F3-Cys Peptide Conjugate ([^125^I]IBMF3)

A solution of 4.6 mCi of Na^125^I in 0.01 N NaOH in a 2 mL glass vial was partially neutralized with an equal volume of 3 µM H_3_PO_4_ solution. A solution of the stannyl maleimide compound **8** (40 µg) in CH_3_OH (40 µL) and 10% HOAc in CH_3_OH (40 µL) were then added sequentially to the vial followed by immediate addition of a solution of *N*-chlorosuccinimide (20 µg) in CH_3_OH (20 µL). The reaction was quenched after 5 min with aq. Na_2_S_2_O_5_ solution (20 µL; 20 µg) and purified by HPLC (Method I; Rt of **[^125^I]5** = 15.8 min) to give 4.1 mCi (89% radiochemical yield) of **[^125^I]5** with >99% radiochemical purity.

A solution of 3.9 mCi of **[^125^I]5** in DMSO (100 µL) was treated with a solution of 1 mg (0.3 µmoles) of F3Cys peptide in 600 µL of PBS buffer (pH 7.0) and the reaction mixture was stored overnight at 4°C. The crude product was purified by HPLC (Method II; Rt of [^125^I]IBMF3 = 35.7 min) and the product fraction was collected, diluted with H_2_O (10 mL) and eluted through a Waters C-18 Sep Pak cartridge (pre-conditioned by sequential elution with 10 mL each of 0.1% TFA in CH_3_CN [Eluant A] and 0.1% TFA in H_2_O [Eluant B] mixtures). The Sep Pak was eluted with Eluant A (10 mL) and [^125^I]IBMF3 was subsequently rinsed off the cartridge with 2 mL of Eluant B. The product solution was concentrated to dryness using a nitrogen stream and formulated in PBS∶EtOH [95∶5] to afford 3.2 mCi of [^125^I]IBMF3 (Overall Rad. Yield: 73%; Rad Purity >99%). The specific activity was 1793±164 Ci/mmol (N = 3).

### Cell Culture

A549 (human lung cancer cell line), purchased from American Type Culture Collection (ATCC, Manassas VA) and MDA-MB-435 (human melanoma) cells [31] were cultured in Dulbecco's modified Eagle's medium supplemented with 10% fetal bovine serum, 1% sodium pyruvate and 1% nonessential amino acids at 37°C in a humidified atmosphere containing 5% carbon dioxide. Tumor cells were harvested when they reached near confluence by incubation with 0.05% trypsin-EDTA. Cells were pelleted by centrifugation at 450× g for 5 min and re-suspended in sterile phosphate-buffered saline.

### Fluorescence Microscopy studies using AF532-F3Cys

MDA-MB-435 cells were grown in optically clear bottom dishes (MatTek Corporation, Ashland, MA) and cells were incubated with serum free or 10% serum harboring DMEM media. Cells were then fixed with 3.5% paraformaldehyde followed by treatment with 70% ethanol. AF532-F3Cys (4 µg/2 ml) was added to cells for 2 h. At the end of the incubation cells were washed, counterstained with DAPI (1 µg/ml) and observed under Nikon TE2000U inverted fluorescent microscope. Images were captured using color camera (Nikon, Melvile NY) and MetaMorph version 4.6.5 software (Universal Imaging Downingtown, PA).

### *In Vitro* Cell Uptake Studies with [^125^I]IBMF3

The uptake of [^125^I]IBMF3 was investigated in MDA-MB-435 cells. Cells were seeded in 6-well dishes. For treatment group, cells were incubated for up to 4 h at 37°C with [^125^I]IBMF3 (0.1 µCi per well) and various concentrations of added cold **6** (none [0 mM], 0.5 mM and 1.0 mM). For the control group, cells were similarly treated with [^125^I]IBMF3 but the media was removed immediately after addition of radioligand. Both control and treated cells were washed twice with ice-cold PBS at the end of incubation, cells collected by scraping and assayed for radioactivity using a Gamma Counter.

### Animal Models

Animal protocols were approved by the University Committee on the Use and Care of Animals (UCUCA approval #09583) at the University of Michigan. Prior to use, MDA-MB-435 cells were trypsinized, counted and suspended in serum-free DMEM media for tumor implantation. Six- to eight-week old *nu/nu* athymic mice were maintained in ventilated cages and fed/watered *ad libitum* and the experiments were carried out under an UCUCA approved protocol as well as following University of Michigan guidelines for the humane use of animals in research. Using sterile surgical scissors, an inverted Y-shaped skin incision over the lower abdomen was made and the skin was retracted with sterile forceps to expose the mammary fat pads. MDA-MB-435 tumor cells (2×10^6^) were then injected in a total volume of 50 µl using a 22 gauge needle. The surgical incision was closed with Vetbond and the rate of tumor growth was monitored over a 2–3 week period using caliper measurements of tumor cross diameters to provide tumor volumes. For Fluorescence imaging MDA-MB-435 and A549 xenografts were grown subcutaneously (s.c.) in the lower flank of the mice. Animals developed tumors after 14–21 days. For *ex vivo* analysis, animals were sacrificed 2 h after administration of peptide and tumor and excretory organs were harvested. Fluorescence was monitored as described below in *in vivo* fluorescence imaging.

### *In Vivo* Fluorescence Imaging

Mice with established subcutaneous MDA-MB-435 tumors in the flanks were intravenously injected with 50 ul (1 mg/ml solution in normal saline containing 5% ethanol) of either AF647-F3Cys or AF647-Control peptide. *In vivo* fluorescence imaging was carried out using an IVIS Spectrum (Caliper Life Science, Hopkinton, MA) imaging system with 640 nm excitation and 680 nm emission band-pass filters and epi-illumination excitation light. Animals were imaged at 5 min, 1 h, 4 h and 24 h post-injection.

### SPECT/CT Imaging

Mouse SPECT/CT imaging studies were performed on a GMI Tri-Modality CT/PET/SPECT small animal scanner (Gamma-Medica Ideas, Inc., Northridge, CA). SPECT images were acquired using a dual-head, high-resolution, low-energy parallel-hole collimator. CT imaging of animals was performed for anatomical co-registration and CT fusion of SPECT images were performed using AMIRA (version 3.1) software (Visage Imaging, San Diego, CA USA). Imaging was performed when tumors reached a 60–100 mm^3^ average volume. Animals under isofluorane anesthesia were injected via the tail vein with [^125^I]IBMF3 (570–610 µCi in 100 µL of PBS∶EtOH [95∶5]) and a series of image acquisitions were obtained at 5 min, 30 min, 1 h, 2 h, 4 h and 24 h post-injection.

### Biodistribution Studies

Female CD-1 nude mice (nu/nu, 25–28 g) bearing MDA-MB-435 mammary fat pad tumor xenografts on opposite sides were utilized for the biodistribution studies. Four animals (N = 4) were used per time point. The radioligand (7.8–10 µCi of [^125^I]IBMF3 in 100 µL of PBS∶EtOH [95∶5]) was injected intravenously and animals were sacrificed by cervical dislocation at designated time intervals (5 min, 30 min, 1 h and 4 h post-injection). Selected tissues (blood, heart, liver, lung, spleen, adrenals, tumor, kidney, muscle, whole brain and thyroid) were harvested and tissue samples were assayed for activity using a Gamma Counter. Tissue uptake was computed as % injected dose/gram (%ID/g).

### Analysis of Mouse Blood and Urine Metabolites of [^125^I]IBMF3

Female, athymic nude mice (N = 2) bearing MDA-MB-435 tumors were injected intravenously with approximately 570–610 µCi of [^125^I]IBMF3 in 100 µL of PBS∶EtOH [95∶5] and sacrificed 5 min later. Blood and urine samples were collected, centrifuged at 12,000 rpm for 15 min, and the supernatants transferred to a fresh centrifuge tube and the centrifugation repeated. The supernatants were treated with CH_3_CN and centrifuged again to precipitate proteins and the clear supernatants analyzed by radio-HPLC (Method I). The percentages of [^125^I]IBMF3 and radiometabolites were determined by measuring the radioactivity of the respective isolated HPLC fractions using a calibrated Gamma Counter and also by integration of their respective HPLC peak areas.

## Supporting Information

Table S1 **Biodistribution of** ***i.v.*** **administered [^125^I]IBMF3 in selected tissues at various time points post injection.** Mice (n = 4 per time interval) were injected via tail vein with [^125^I]IBMF3 and tissues were harvested for radioactivity uptake. Distribution of [^125^I]IBMF3 at various time points post injection was monitored by determining the radioactivity in the different tissues and tabulating them as percent injected dose per gram of tissue. All the data are computed as mean +/− SEM.
(DOCX)
