# Supplementary material for: Synthesis and Investigation of a Radioiodinated F3 Peptide Analog as a SPECT Tumor Imaging Radioligand
Source: PLoS One. 2011 Jul 19;6(7):e22418. doi: 10.1371/journal.pone.0022418 (PMC3139646; doi:10.1371/journal.pone.0022418)
Supplement: Table S1 — Biodistribution of i.v. administered [125I]IBMF3 in selected tissues at various time points post injection. Mice (n = 4 per time interval) were injected via tail vein with [125I]IBMF3 and tissues were harvested for radioactivity uptake. Distribution of [125I]IBMF3 at various time points post injection was monitored by determining the radioactivity in the different tissues and tabulating them as percent injected dose per gram of tissue. All the data are computed as mean +/− SEM. (DOCX) [file pone.0022418.s001.docx]

**Table S1**

|  | **5 min** | **30 min** | **60 min** | **240 min** |
| --- | --- | --- | --- | --- |
| **Tumor** | 0.3±0.11 | 1.05±0.45 | 0.62±.031 | 0.13±0.03 |
| **Blood** | 0.7±0.09 | 0.84±0.25 | 0.30±0.09 | 0.12±0.02 |
| **Muscle** | 0.14±0.06 | 0.38±0.04 | 0.14±0.08 | 0.04±0.01 |
| **Liver** | 3.76±4.64 | 1.86±0.27 | 0.78±0.20 | 0.21±0.11 |
| **Adrenal** | 0.25±.12 | 0.36±0.30 | 0.12±0.01 | 0.07±0.01 |
| **Spleen** | 0.19±0.03 | 0.46±0.16 | 0.15±0.06 | 0.10±0.03 |
| **Kidney** | 5.68±1.06 | 10.78±3.4 | 2.9±1.72 | 0.45±0.11 |
| **Lung** | 0.53±0.53 | 0.82±0.64 | 0.33±0.32 | 0.52±0.34 |
| **Heart** | 0.24±0.04 | 0.39±0.12 | 0.12±0.05 | 0.04±0.01 |
| **Thyroid** | 0.26±0.01 | 2.60±1.33 | 1.67±1.14 | 4.04±2.25 |
